# Efficient implementation of LMS adaptive filter-based FECG extraction on an FPGA

**DOI:** 10.1049/htl.2020.0016

**Published:** 2020-11-13

**Authors:** Bhavya Vasudeva, Puneesh Deora, Pradhan Mohan Pradhan, Sudeb Dasgupta

**Affiliations:** Department of Electronics and Communication Engineering, Indian Institute of Technology Roorkee, Uttarakhand, India

**Keywords:** electrocardiography, least mean squares methods, medical signal processing, adaptive filters, flip-flops, field programmable gate arrays, obstetrics, series architecture, existing FPGA implementations, FECG extraction methods, LMS adaptive filter-based FECG extraction, FPGA implementation, foetal heart rate monitoring system, preprocessing unit, foetal electrocardiogram extraction unit, FHR detection unit, arithmetic operations, floating-point unit, mean squares, LMS-AF, lower utilisation, parallel architecture, convergence time, extracted FECG, noninvasive FECG databases

## Abstract

In this Letter, the field programmable gate array (FPGA) implementation of a foetal heart rate (FHR) monitoring system is presented. The system comprises a preprocessing unit to remove various types of noise, followed by a foetal electrocardiogram (FECG) extraction unit and an FHR detection unit. To improve the precision and accuracy of the arithmetic operations, a floating-point unit is developed. A least mean squares algorithm-based adaptive filter (LMS-AF) is used for FECG extraction. Two different architectures, namely series and parallel, are proposed for the LMS-AF, with the series architecture targeting lower utilisation of hardware resources, and the parallel architecture enabling less convergence time and lower power consumption. The results show that it effectively detects the R peaks in the extracted FECG with a sensitivity of 95.74–100% and a specificity of 100%. The parallel architecture shows up to an 85.88% reduction in the convergence time for non-invasive FECG databases while the series architecture shows a 27.41% reduction in the number of flip flops used when compared with the existing FPGA implementations of various FECG extraction methods. It also shows an increase of 2–7.51% in accuracy when compared to previous works.

## 1 Introduction

Over the past few decades, analysis of foetal electrocardiogram (FECG) has proven to be a tool of great importance when it comes to monitoring the well-being of the foetus during pregnancy and labour, unearthing vital information such as foetal heart rate (FHR), heart rate variability etc. Any abnormalities in these parameters indicate that the foetus is in distress, possibly due to asphyxia, which is a major cause of neonatal deaths. Regular FHR monitoring can enable a clinician to intervene in due time to prevent such cases.

Various methods [1] used to obtain FHR are auscultation (Doppler ultrasound, fetoscope), foetal phonocardiography, foetal magnetocardiography, and other invasive methods where electrodes have direct contact with foetal skin. These methods are not suitable for mobile, regular, low-cost, real-time monitoring of the foetus. An alternative method to obtain FHR is to calculate it from FECG, which can be extracted from the non-invasive abdominal electrocardiogram (ECG) recordings acquired from a pregnant subject. This ECG signal contains FECG contaminated with maternal ECG (MECG), power line interference, motion artefacts etc. Various techniques [1] involving statistical and time-domain analysis have been exploited to extract the FECG. Adaptive filtering [2], non-linear decomposition [3], blind source separation [4] (independent component analysis (ICA)-based methods), wavelet transform (WT)-based techniques [5–7], neural network-based approaches [8] are widely used.

Although the methods based on ICA perform better than those which use single-channel recordings, they require the acquisition of multi-channel abdominal signals which may be uncomfortable for the subject. Such methods also require visual inspection of the signals, and an appropriate number of data segments have to be selected manually for a representative template [1]. The real-time implementation of such methods is not suitable unless block delayed analysis is considered [9]. Among the methods using single-channel recordings, Kalman filter-based approaches offer high performance, but their implementation complexity is quite high and they require the R peaks of the signal to have a consistent morphological shape [9]. WT-based methods can also prove to be computationally intensive, can have drawbacks such as increased hardware area, complex routing etc., and require the selection of an appropriate wavelet, to achieve the required performance level. As foetal signals and other interferences are not always linearly separable, non-linear decomposition-based methods can be used for extracting FECG in such cases. However, such methods require some prior information about the desired and undesired parts of the signal and have a high-computational complexity [9]. As the adaptive filter is an accurate method for FECG extraction and its computational complexity is relatively low [1], a least mean squares algorithm-based adaptive filter (LMS-AF) is chosen for this study.

The signal strength of the FECG is low as compared to the MECG [9]. In rural areas, where cellular connectivity is low, the transmission of these signals for processing in the cloud is not suitable. The FPGA can eliminate the need for an extra standby computing device that would be required for computational purposes. Also, it is a better prototyping platform for hardware implementation compared to traditional digital signal processors (DSPs). The FPGA implementation can also serve as a step towards the development of a low-cost FHR monitoring system as a system on chip.

Previous hardware implementations of LMS-AF focusing on FECG extraction are discussed below. The work presented by Hatai *et al.* [10] was tested on synthetic data, with up to 88% accuracy. As synthetic data has significantly better morphology of the PQRST complex and lower noise than real signals, no preprocessing was performed. Dynamic thresholding was used for peak detection. Morales *et al.* [11] used a field-programmable analogue array for analogue signal preprocessing and an FPGA for FECG extraction with accuracy in the range of 87–93%. LabVIEW FPGA module was used to generate the hardware design. Arias-Ortega *et al.* [12] implemented the LMS-AF on a digital signal controller, used a low-noise analogue front end to achieve 93.1% accuracy and 87.1% sensitivity. In [13], the authors implemented an LMS-AF-based FECG extraction system on an FPGA. However, FHR calculation is done manually and an algorithm to automate this process is not presented. Some other methods for FECG extraction [14–18] have also been implemented on hardware. Some of the aforementioned works [11, 15, 16] reportedly use fixed-point arithmetic, which leads to lower precision than floating-point (FP) arithmetic. Furthermore, FP addition utilises much fewer resources than the logarithmic number system (LNS). As the main advantage of the LNS is its efficient division operation, which is not required in LMS-AF, we have opted for LMS with FP operations. It has also been reported that FP operations are difficult to implement on FPGA as the algorithm is very complex and leads to excessive consumption of logic elements [19].

The main contributions of this Letter are as follows:

- For the foetal R peak detection, a norm for the determination of the threshold is proposed to avoid the detection of false positives.
- A FP unit (FPU) is developed for the FPGA implementation to support FP calculations, and hence improve the precision and accuracy of the system. Although Xilinx has a core for FP operations, it is not an open-source internet protocol, and thus cannot be used for application-specific integrated circuit designing, which is why the FPU is developed. The system is tested on both real and synthetic ECG signals, and the results validate the robustness of the system.
- For the implementation of the LMS-AF module, two different architectures, namely series and parallel, are proposed. While the former is developed for lower hardware utilisation, the latter is better in terms of lower latency and power consumption. FPGA implementation and simulation results validate the same.

## 2 Methodology

### 2.1 Preprocessing

The information that needs to be extracted from the thoracic and abdominal signals is masked by various types of noise [9], such as power line interference at 50 Hz, low-frequency baseline wander, broadband muscle noise, motion artefact etc. To retain the MECG and FECG components [9] and attenuate the sources of noise, the signals are preprocessed.

To remove the high frequencies, a fourth-order low-pass Butterworth filter is used. The cut-off of the filter is kept at 45 Hz so that the ECG components in the signal are retained [9]. For the low-pass filter, the Bessel, Butterworth, Chebyshev, and RC filters [20] were considered. Among these, the Bessel filter offers a slower transition from pass-band to stop-band as compared to the other filters of the same order. The Chebyshev filters have ripple in the pass-band, while Butterworth and Bessel filters do not. Moreover, Butterworth filters have a significantly better frequency response (flat in the pass-band) than a simple RC filter of the same order. Therefore, a Butterworth filter is used in this work. As the cut-off of the filter is not sharp, the frequencies above 35 Hz and below 55 Hz lie in the transition band of the filter.

The peak at 50 Hz due to the power line interference is not sufficiently attenuated by the low-pass filter. Therefore, a notch filter [20] centred at 50 Hz (quality factor 25) is used.

Another source of noise is the baseline wander, which is a low-frequency noise is resulting from the respiration or movement of the subject or electrodes during recording. Since only the components corresponding to the baseline wander need to be removed, a high-pass filter is not used for this application. Three techniques, namely polynomial fitting using polynomial regression, two-stage median filtering, and two-stage moving average filtering [21], are considered to obtain an approximation of the baseline wander present in the signal. The complexity of these methods is $O\lpar mN^2\rpar $, $O\lpar Nn\log \lpar n\rpar \rpar $, and $O\lpar N\rpar $, respectively, where *N* is the total number of samples, *m* is the order of the polynomial, and *n* is the window size. A two-stage moving average filter is used in this work as it is the most efficient of all and gives a smooth approximation of the baseline wander. The operations performed are summarised in the following equations:

(1) $$M_1\lsqb n\rsqb = \displaystyle{1 \over {N_1}}\sum\limits_{i = 0}^{N_1 - 1} x\lsqb n + i - N_1 + 1\rsqb \eqno\lpar 1\rpar $$

(2) $$M_2\lsqb n\rsqb = \displaystyle{1 \over {N_2}}\sum\limits_{\,j = 0}^{N_2 - 1} M_1\lsqb n + j - N_2 + 1\rsqb \eqno\lpar 2\rpar $$

where *x* is the input signal, $M_1$ is the first stage mean with window size $N_1$, $M_2$ is the second stage mean with window size $N_2$, and *n* is the sample index. $N_1$ and $N_2$ are kept as 200 in this work as a larger value yields an extremely smooth estimate, due to which some of the changes in the baseline are not captured properly, whereas a smaller value yields an erratic estimate. To remove the baseline wander, the output of the two-stage moving average filter is subsequently subtracted from the input signal.

### 2.2 LMS algorithm

To separate the FECG from the preprocessed thoracic and abdominal ECG signals, LMS-AF [22] is used. Let ${\bi x}\lsqb n\rsqb = \lsqb x\lsqb n\rsqb \comma \; x\lsqb n - 1\rsqb \comma \; \ldots \comma \; x\lsqb n - m + 1\rsqb \rsqb ^{\rm T}$, represent the input to the filter, where $x\lsqb n\rsqb $ is the sample value at instant *n*, *m* is the order of the filter, and $\lpar .\rpar ^{\rm T}$ denotes the transpose operator. ${\bi w}\lsqb n\rsqb = \lsqb w_{m - 1}\lsqb n\rsqb \comma \; w_{m - 2}\lsqb n\rsqb \comma \; \ldots \comma \; w_0\lsqb n\rsqb \rsqb $, is the weight vector, where $w_{m - k}\lsqb n\rsqb $ is the *k*^th^ weight at sample instant *n*. The output of the filter at the *n*^th^ sampling instant is given by (3). The error signal is calculated using (4), where $d\lsqb n\rsqb $ is the desired signal. The weight updation is carried out using (5), where $\mu $ is the step size, $\nabla $ is the gradient operator, and $k = 0\comma \; 1\comma \; \ldots \comma \; m - 1$.

(3) $$y\lsqb n\rsqb = {\bi x}^{\rm T}\lsqb n\rsqb {\bi w}\lsqb n\rsqb \eqno\lpar 3\rpar $$

(4) $$e\lsqb n\rsqb = d\lsqb n\rsqb - y\lsqb n\rsqb \eqno\lpar 4\rpar $$

(5) $$\eqalign{w_k\lsqb n + 1\rsqb & = w_k\lsqb n\rsqb - \mu \nabla \lpar e^2\lsqb n\rsqb \rpar \cr & = w_k\lsqb n\rsqb + 2\mu e\lsqb n\rsqb x\lsqb n - m + k + 1\rsqb } \eqno\lpar 5\rpar $$

For this work, the thoracic signal is considered as the desired signal $d\lsqb n\rsqb $, and the abdominal signal is the input ${\bi x}\lsqb n\rsqb $. The criteria for convergence of the filter weights are satisfied in around 12,000 samples. *m* and $\mu $ are set to 19 and $7 \times 10^{ - 5}$, respectively.

The MECG components in thoracic and abdominal signals are not exactly the same [1], which leads to some residual maternal R peaks in the resulting error signal $e\lsqb n\rsqb $ [22]. After the convergence of weights of the filter, the FECG is enhanced and the MECG is attenuated in $e\lsqb n\rsqb $ (the output of LMS-AF).

### 2.3 FHR detection

The Pan and Tompkins algorithm (PTA) [23] is well-known for detecting R peaks in the ECG signals of a single subject. A modified version of PTA is used to detect the foetal R peaks from a mixture of foetal R peaks and residual maternal R peaks. The output of the LMS-AF is differentiated, squared, and then passed through a mean filter of length 40. Since the extracted FECG contains residual maternal R peaks as well as sharper foetal R peaks, these operations, which are also a part of PTA, enhance the foetal R peaks. Hence, the resultant signal sdm has higher amplitude for foetal R peaks as compared to the maternal R peaks. To determine the threshold value *th* which can be used to distinguish between the foetal and maternal R peaks, a new norm is proposed. Unlike PTA where adaptive thresholding is used, the proposed method uses a single value of ‘*th*’ for a data set. $m_1$ denotes the mean of the signal ‘*sdm*’. The procedure for calculation of ‘*th*’, which is repeated *N* times, is summarised below:

(1) If (${in} \gt m_1$ and ${in} \gt R_1$) then $R_3 = {in}\comma \; R_4 = R_2$
(2) else if (${in} \lt m_1$) then ${pv} = R_3\comma \; {pl} = R_4\comma \; m_2 = m_2 + \displaystyle{{{pv}} \over N}$
(3) end if
(4) $R_1 = {in}$
(5) $R_2 = R_2 + 1$
(6) If ($R_2 = N - 1$) then ${th} = \lpar m_1 + m_2\rpar /2$
(7) end if

Here, *in* denotes the current input, *N* is the number of inputs, $R_1\left({R_2} \right)$ is used to store the input value (location) for the next cycle, and $R_3\left({R_4} \right)$ is used to conditionally store the input value (location). The locations and values of local maxima are denoted by *pl* and *pv*, respectively. $m_1$, $m_2$, $R_1$, and $R_2$ are initialised to zero.

The detection of foetal R peaks is based on two criteria: their amplitude should be above ‘*th*’, and if two local maxima occur within 200 samples of each other, the one with the larger amplitude denotes the foetal R peak. The maximum FHR can be 200 beats per minute (bpm) [24] (300 samples at 1 kHz sampling frequency). Therefore, maxima separated by at least 200 samples (300 bpm) are considered for the detection of foetal R peaks. The procedure for the above is listed below:

(1) if (${pv} \gt { th}$) then
(2)  if (${pl} - R_1 \gt 200$) then ${out} = R_1\comma \; R_1 = {pl}\comma \; R_2 = {pv}$
(3)  else
(4)   if (${pv} \gt R_2$) then ${out} = {pl}\comma \; R_1 = {pl}\comma \; R_2 = {pv}$
(5)   else ${out} = R_1$
(6)    end if
(7)   end if
(8)  end if

Here, out denotes the locations of the foetal R peaks detected. $R_1$ and $R_2$ are initialised with *pl* and *pv*, respectively.

The difference between the consecutive R peaks, as detected in the previous stage, is the RR interval. As the weights of the LMS-AF converge around 12,000 samples, only the RR intervals for peaks occurring after 12,000 samples are considered. The average of these RR intervals is taken and divided by the sampling frequency to get the average RR interval length in seconds. The FHR is calculated as follows:

(6) $${\rm FHR}\lpar {\rm bpm}\rpar = \displaystyle{{60} \over {{\rm RR}\, {\rm interval}\, {\rm length}\, \lpar {\rm s}\rpar }}\eqno\lpar 6\rpar $$

## 3 Implementation of FPGA

**Fig. 1:**
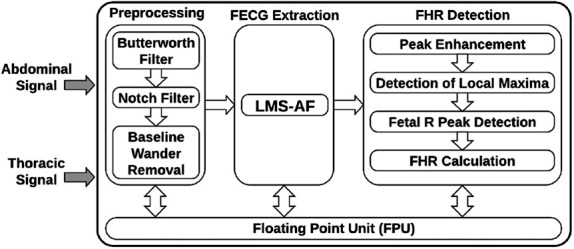
Block diagram of the FPGA implementation of the system

For the FPGA implementation, the proposed system is divided into four units as shown in Fig. 1.

### 3.1 FP unit

Since the input values to the system are FP numbers, logic cannot be defined directly on such numbers in Verilog. Therefore, an FPU is developed for performing basic arithmetic operations (addition, subtraction, and multiplication) and comparison. The FP numbers are converted to their 32-bit binary representation as per the IEEE 754 standard [25], in which the first bit is the sign bit *s*, followed by 8 bits for exponent *e*, and 23 bits for fraction *f*. The value of the number is given by $\lpar - 1\rpar ^s 1\cdot f 2^{\lpar e - {\rm bias}\rpar }$, where $1\cdot f$ is the mantissa *m* and *bias* is $2^7 - 1$. The inputs to the FPU module include a 2-bit sequence to select one of the four available operations and two 32-bit FP numbers (*A* and *B*). When the numbers enter the module, they are split into three parts, i.e. sign, exponent and mantissa denoted by $s_a$, $e_a$, $m_a$ and $s_b$, $e_b$, $m_b$ for *A* and *B*, respectively. *m* is stored in the form of 24 bits with 1 concatenated to 23 bits of *f*. $s_{{\rm out}}$, $e_{{\rm out}}$, and $m_{{\rm out}}$ denote the sign, exponent and mantissa of the output.

The procedure followed for the FP adder is listed below:

(1) if ($e_a = e_b$) then $e_{{\rm out}} = e_a$
(2) else if ($e_a \gt e_b$) then $e_{{\rm out}} = e_a$, $d = e_a - e_b$, $m_b = m_b \gg d$
(3) else $e_{{\rm out}} = e_b$, $d = e_b - e_a$, $m_a = m_a \gg d$
(4) end if
(5) if ($s_a = s_b$) then $m_{{\rm out}} = m_a + m_b$, $s_{{\rm out}} = s_a$
(6) else
(7)   if ($m_a \gt m_b$) then $s_{{\rm out}} = s_a$, $m_{{\rm out}} = m_a - m_b$
(8)   else $s_{{\rm out}} = s_b$, $m_{{\rm out}} = m_b - m_a$
(9)   end if
(10) end if

Here, $ \gg $ denotes the right shift operation. A similar procedure is followed for the FP subtractor, except that when the sign bits are same, subtraction is performed after comparing the mantissas and when they are opposite, addition is performed. The outputs of the FP multiplier are given by $s_{{\rm out}} = s_a \oplus s_b$, $e_{{\rm out}} = e_a + e_b - {bias}$, and $m_{{\rm out}} = m_a \times m_b$, where $ \oplus $ denotes the bit-wise XOR operation. For all the three operations, the next stage is to normalise the output. When $m_{{\rm out}}$ is not of the form $1\cdot f_{{\rm out}}$, a repetitive process of shifting $m_{{\rm out}}$ left by one place and subtracting 1 from $e_{{\rm out}}$ is followed till the first bit of $m_{{\rm out}}$ becomes 1. $s_{{\rm out}}$, $e_{{\rm out}}$, and $f_{{\rm out}}$ are concatenated to get the 32-bit output of the operation.

For FP comparison, let $c_{{\rm out}}$ denote a 2-bit sequence to denote the three cases, i.e. $A \gt B\ \lpar c_{{\rm out}} = 01\rpar $, $A = B\ \lpar c_{{\rm out}} = 00\rpar $, and $A \lt B\ \lpar c_{{\rm out}} = 10\rpar $. The procedure is listed below:

(1) If ($s_a \gt s_b$) then $c_{{\rm out}} = \lsqb 10\rsqb $
(2) else if ($s_b \gt s_a$) then $c_{{\rm out}} = \lsqb 01\rsqb $
(3) else
(4)   if ($e_a \gt e_b$) then $c_{{\rm out}} = \lsqb 01\rsqb $
(5)   else if ($e_b \gt e_a$) then $c_{{\rm out}} = \lsqb 10\rsqb $
(6)   else
(7)    if ($f_a \gt f_b$) then $c_{{\rm out}} = \lsqb 01\rsqb $
(8)    else if ($f_b \gt f_a$) then $c_{{\rm out}} = \lsqb 10\rsqb $
(9)    else $c_{{\rm out}} = \lsqb 00\rsqb $
(10)    end if
(11)   end if
(12) end if

As the output should be a 32-bit number for uniformity, 30 $0s$ are concatenated to $c_{{\rm out}}$ to get the output.

### 3.2 Preprocessing: The following three modules are implemented

#### 3.2.1 Butterworth filter

In this module, one input value is used in every clock cycle to get the output as follows [20]:

$$\eqalign{O\lsqb k\rsqb & = \alpha I\lsqb k\rsqb + \beta O\lsqb k - 1\rsqb + \gamma O\lsqb k - 2\rsqb + \delta O\lsqb k - 3\rsqb \cr &\quad + \epsilon O\lsqb k - 4\rsqb }$$

where $I\lsqb k\rsqb $ is the sample value at instant *k* and $O\lsqb k\rsqb $ is the output value. The values of the constants are obtained from the transfer function of the filter, as per the cut-off and order of the filter. In this work, $\alpha = 0.00308$, $\beta = 3.28391$, $\gamma = - 4.08689$, $\delta = 2.28117$, and $\epsilon = - 0.48140$.

#### 3.2.2 Notch filter

This module works similarly to the previous module, following the equation [20]:

$$O\lsqb k\rsqb = \alpha I\lsqb k\rsqb + \beta I\lsqb k - 1\rsqb + \gamma I\lsqb k - 2\rsqb + \delta O\lsqb k - 1\rsqb + \epsilon O\lsqb k - 2\rsqb $$

In this case, $\alpha = 0.99405$, $\beta = - 1.31278$, $\gamma = 0.99405$, $\delta = 1.31272$, and $\epsilon = - 0.98804$.

#### 3.2.3 Baseline wander removal

**Fig. 2:**
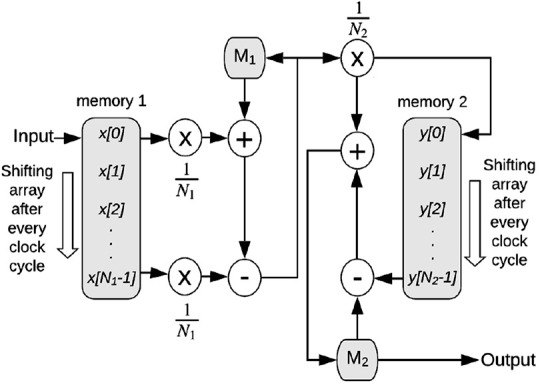
Structure of the two-stage moving average filter

Fig. 2 shows the structure of the two-stage moving average filter. As in (1), the first stage mean $M_1$ is the average of $N_1$ values. In every clock cycle, the input is added to $M_1$ and $x\lsqb N_1 - 1\rsqb $ is subtracted from $M_1$, both after getting multiplied by $\left({1/N_1} \right)$. For the moving average operation, all the values in memory 1 are shifted by one position, so that $x\lsqb N_1 - 1\rsqb $ is discarded and a new value is stored in $x\lsqb 0\rsqb $. A similar procedure is followed for calculating the second stage mean $M_2$ as per (2). $M_1$ is multiplied by $\left({1/N_1} \right)$, stored in memory 2 and also added to $M_2$. The last value of memory 2 can then be directly subtracted from $M_2$ to obtain the second stage mean. The values of $M_2$ represent the baseline wander approximation. The output of the two-stage moving average filter is used to remove the baseline wander from the input by performing one subtraction operation in every clock cycle. The latency of each of these modules is 1 clock cycle.

### 3.3 FECG extraction

**Fig. 3:**
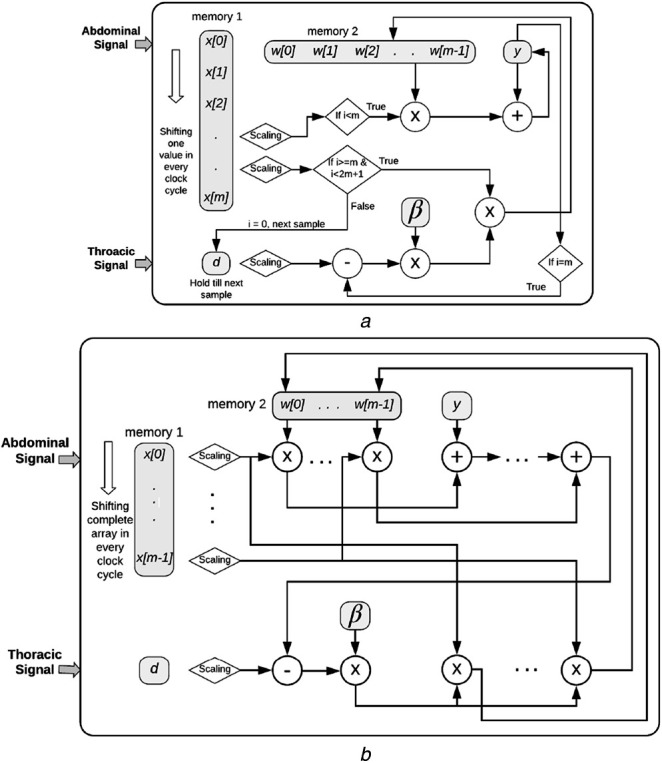
Illustration of proposed series and parallel architecture of LMS-AF *a* Series architecture *b* Parallel architecture

This stage comprises the LMS-AF, for which two different architectures are proposed. Figs. 3*a* and *b* illustrate the proposed series and parallel architectures of the LMS-AF module, respectively. In both the figures, $\beta = 2\mu $. As the magnitude of abdominal and thoracic signals may not be of the same order, they have to be scaled appropriately (denoted by scaling in the figures) before being used in the algorithm.

#### 3.3.1 Series architecture

In Fig. 3*a*, memory 1 stores the vector ${\bi x}^{\rm T}\lsqb n\rsqb $ and an extra element, and memory 2 contains the weights of the filter. In every clock cycle, one element of $x^{\rm T}\lsqb n\rsqb $ is scaled, multiplied by one element of ${\bi w}\lsqb n\rsqb $ and added to $y\lsqb n\rsqb $. The same element of ${\bi x}^{\rm T}\lsqb n\rsqb $ is also copied to the immediate next position in ${\bi x}^{\rm T}\lsqb n\rsqb $. Thus, after *m* clock cycles, $y\lsqb n\rsqb $ has been obtained as in (3), and ${\bi x}^{\rm T}\lsqb n\rsqb $ has shifted by one index. In the following clock cycle, error is calculated using (4), and the updated value of the first weight of the filter is also obtained. This updated weight value is stored in its position in the next clock cycle. This sequential process is repeated until all the weights are updated, which corresponds to $m + 1$ clock cycles. After a total of $2m + 1$ clock cycles, a new input value is stored in $x\lsqb 0\rsqb $ so that ${\bi x}^{\rm T}\lsqb n\rsqb $ is updated. The register containing $d\lsqb n\rsqb $ also gets updated. As the required output for a particular pair of ${\bi x}^{\rm T}\lsqb n\rsqb $ and $d\lsqb n\rsqb $ is obtained after $2m + 1$ clock cycles, the latency of this module is $2m + 1$ clock cycles.

#### 3.3.2 Parallel architecture

In Fig. 3*b*, the memory 1 (vector ${\bi x}^{\rm T}\lsqb n\rsqb $) gets updated with the next input value in every clock cycle. Each element of ${\bi x}^{\rm T}\lsqb n\rsqb $ is scaled, and then multiplied with the elements from the memory 2 (vector ${\bi w}\lsqb n\rsqb $). These are added to obtain $y\lsqb n\rsqb $, as in (3). The register containing $d\lsqb n\rsqb $ is updated in every clock cycle and is used to calculate the error, using (4). Since $2\mu e\lsqb n\rsqb $ is used in every weight updation, it is calculated first, and subsequently multiplied with the values from memory 1 to update the weights, using (5). The updated weights are stored in memory 2. Thus, all the operations involving the error calculation and weight updation are performed in a single clock cycle and the latency of this module is 1 clock cycle.

### 3.4 FHR detection: The four modules of this stage are

#### 3.4.1 Peak enhancement

In this module, the input is differentiated, squared, and passed through a mean filter of length *P*. The mean $m_1$ of *sdm*, which is required in the next module, is also determined in this module. The operations executed in every clock cycle are summarised below:

1. ${pval} = {cval}$
2. ${cval} = {input}$
3. ${sdiff} = \lpar {cval} - {pval}\rpar \times \lpar {cval} - {pval}\rpar $
4. $M\lsqb 0\rsqb = {sdiff} \times \displaystyle{1 \over P}$
5. ${sdm} = {sdm} + M\lsqb 0\rsqb - M\lsqb P - 1\rsqb $
6. $m_1 = m_1 + {sdm} \times \displaystyle{1 \over N}$
7. Shift the elements of *M* by one position

Here, *cval* and *pval* denote the current and previous input values, respectively. *sdiff* denotes the differentiated and squared signal, which is stored in the memory *M* (size *P*) after multiplication by 1/*P*. *N* denotes the number of input samples.

#### 3.4.2 Detection of local maxima

In this module, the local maxima are determined, using $m_1$ as the threshold. The operations executed in every clock cycle are listed in Section 2.3.

#### 3.4.3 Foetal R peak detection

The operations executed in every clock cycle for this module are also listed in Section 2.3.

#### 3.4.4 FHR calculation

The RR intervals are estimated using the differences between consecutive peak locations (*out*). Two registers are used for storing the current input and the previous input. The estimated RR intervals are accumulated and averaged out, after which FHR is obtained using (6).

## 4 Results and discussion

**Fig. 4:**
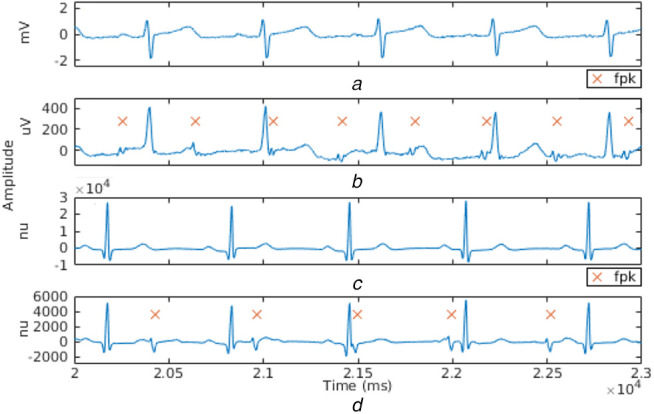
Waveforms representing various ECG signals *a* Real thoracic signal *b* Real abdominal signal *c* Synthetic thoracic signal *d* Synthetic abdominal signal. Synthetic data set has no units (nu)

To test the system for real signals, the non-invasive FECG (NiFECG) database [26] and database for identification of systems (DaISy) [27] are used. In the NiFECG database, the signals have been sampled at 1 kHz with 16-bit resolution. From the data set to be tested, one thoracic and one abdominal signal are chosen as inputs to the system. These are shown in Figs. 4*a* and *b*, respectively. DaISy consists of eight channels, 10 s recordings sampled at 250 Hz, where three channels are thoracic signals and the rest are abdominal signals. The synthetic signals were simulated using the FECGSYN toolbox [28] in MATLAB, at a sampling rate of 1 kHz. Figs. 4*c* and *d* show the thoracic and abdominal input signals. It is observed that the synthetic signals are less noisy than the real signals. In the thoracic signals, all the peaks are maternal R peaks. In the abdominal signals, the higher peaks are maternal, and those annotated as *fpk* are foetal R peaks.

**Fig. 5:**
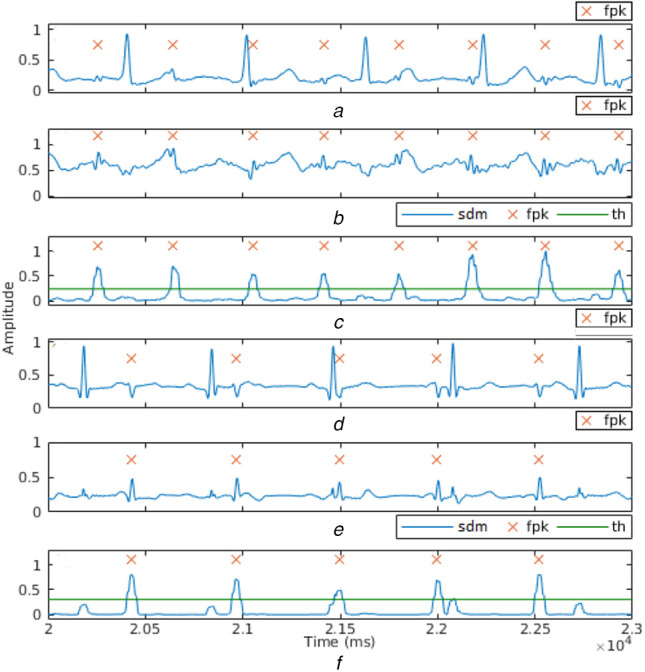
Results of *a* Preprocessing *b* LMS-AF *c* Peak detection for real signals *d* Preprocessing *e* LMS-AF *f* Peak detection for synthetic signals. All values are normalised between 0 and 1

Figs. 5*a* and *d* show the time series for real and synthetic signals after preprocessing. The output of the LMS-AF stage, where FECG is enhanced and MECG is attenuated, is shown in Figs. 5*b* and *e*. The signal obtained after peak enhancement (labelled *sdm*) and the detected foetal R peaks (denoted by *fpk*), are shown in Figs. 5*c* and *f*. Table 1 lists the results obtained for sensitivity, specificity, accuracy, and FHR for the tested datasets. It is observed that the proposed norm for the determination of the threshold, which is used for foetal R peak detection, results in no false positives. The application of PTA [23] on ecga444 [26] data set gives a sensitivity of 78.72%, specificity of 48.28%, and accuracy of 67.11%, as this method was developed for detecting R peaks in ECG signals of a single subject, whereas the output of LMS-AF has enhanced FECG as well as residual MECG.

**Table 1:** Results obtained for different datasets using the proposed approach

Dataset	FHR, bpm	Sensitivity, %	Specificity, %	Accuracy, %
ecgca444 [26]	152	95.74	100	97.37
ecgca840 [26]	161	96	100	97.37
ecgca746 [26]	147	97.78	100	98.53
ecgca771 [26]	153	100	100	100
DaISy Channel 2 [27]	143	100	100	100
DaISy Channel 3 [27]	143	100	100	100
synthetic [28]	115	100	100	100

In Table 2, the performance of the proposed work is compared with various FECG extraction methods. The proposed work shows an increase of 1.34% in the sensitivity and 2% in the accuracy for DaISy. The proposed method also shows an increase of 1.02% in the sensitivity and 7.51% in accuracy when compared to works that have tested their systems on both NiFECG and DaISy.

**Table 2:** Comparison of performance of the proposed method with various FECG extraction methods

Method	Dataset	Sensitivity, %	Accuracy, %
Le *et al.* [29]	DaISy	98.68%	98.04
Gini *et al.* [30]	DaISy	91%	87.30
Lima-Herrera *et al.* [31]	DaISy and NiFECG	97.50%	92.10
Morales *et al.* [11]	DaISy and NiFECG	—	89
proposed method	DaISy	100%	100
proposed method	DaISy and NiFECG	98.5%	99.04

–, Not reported.

The system is implemented on the Xilinx Artix-7 FPGA (XC7A100TCSG324-1), equipped with 63,400 look-up tables (LUTs), 126,800 flip flops (FFs), 240 DSPs, and 210 input/output ports. Except for the baseline wander removal, the detection of local maxima, and the LMS-AF module, all the modules have minimal resource (∼0 LUTs and FFs) and power utilisation (0.068 W). The baseline wander removal module consumes 2.691 W power, and utilises 820 LUTs and 94 FFs. The power per cycle is 89.683 ${\rm \mu W}$. The detection of the local maxima module consumes 0.167 W power, and utilises 45 LUTs and 34 FFs. The power per cycle is 9.278 ${\rm \mu W}$.

For the LMS-AF module, the resource utilisation and power consumption depend on the architecture and the order of the filter. There is a trade-off between the series and parallel architectures in terms of convergence time and resource utilisation. For the parallel design, the number of operations in every clock cycle is more than the series design, and hence the resource utilisation is greater. On the other hand, the series architecture distributes the same number of operations across more clock cycles, and hence needs more time for convergence, and consumes more power. Also, an increase in the filter order results in an increase in the number of operations as well as resource utilisation.

In Table 3, the existing implementations of various FECG extraction methods on different hardware platforms are compared with the proposed architectures of LMS-AF after mapping the power consumption and convergence time to 50 MHz operating frequency. As the number of cycles is different for the two architectures, there is a large difference in the values of power consumption. The power per cycle is 7.823 ${\rm \mu W}$ for series and 65.133 ${\rm \mu W}$ for parallel architecture (30,000 input samples). The series architecture uses nine instances of the FPU module, and shows 27.41% reduction in the number of FFs, whereas the number of LUTs is comparable to the other methods. The parallel architecture uses 98 instances of the FPU module, and shows up to 85.88% reduction in the convergence time when compared with the methods [11, 15, 17] using NiFECG database. The convergence time for series is 39 times $\left({m = 19} \right)$ more than that of parallel architecture, as the latency is $2m + 1$ and 1 clock cycles, respectively.

**Table 3:** Comparison of hardware implementations of the proposed method and various FECG extraction methods

Method	Device	Convergence time, ms	Consumption of power, W	LUTs	FFs
LMS [10]	XC6SLX45-3-CSG394	—	—	1042	440
LMS [11]	Spartan3E XC3S500E	600	—	—	—
LMS [12]	dsPIC30F6014A	0.33	1.67^a^	—	—
OL-JADE [14]	OMAP L137	948	—	—	—
infomax [15]	Stratix-V 5SGXEA7N2F45C2	3.4-54	0.55	—	—
neural network [16]	Stratix-II EP2S15F484C3	—	—	9726	4324
BSS [17]	Spartan-3	—	—	3002	405
proposed series	Artix-7	18.72	6.478	2368	294
proposed parallel	XC7A100TCSG324-1	0.48	1.954	22 407	640

−, Not reported.

^a^The system proposed by Ortega *et al.* [12] consumes 1 W, for the current absorption of 200 mA and supply of 5 V, at 30 MHz operating frequency.

The use of FP operations in the implementation of the proposed architectures greatly enhanced the precision and accuracy of the system. Many of the methods listed in Table 3 have reportedly used fixed-point numbers. The use of fixed-point numbers would have resulted in a lower resource utilisation and power consumption as the operations involving FP numbers are computationally intensive [10, 17, 19]. However, the use of fixed-point numbers compromises with the accuracy of the system.

## 5 Conclusion

In this Letter, the FPGA implementation of a complete system for preprocessing ECG signals, extracting FECG, and subsequently calculating FHR is presented. For the removal of high-frequency components, power line interference, and baseline wander, Butterworth, Notch, and two-stage moving average filters are used, respectively. For FECG extraction, an LMS-AF is used, and series and parallel architectures are designed for its implementation. The precision and accuracy of the complete system are significantly enhanced by the use of FP arithmetic, for which an FPU is developed. Comparisons with previous work show that the proposed parallel architecture requires the least time for convergence of filter weights, while the proposed series architecture has low resource utilisation.
